# Association of serum klotho with cognitive function among individuals with nonalcoholic fatty liver disease

**DOI:** 10.3389/fnagi.2024.1487182

**Published:** 2024-11-05

**Authors:** Feilong Wu, Jie Pan, Mingtao Chen, Xuye Lai, Yingying Gu, Lei Pei, Lili Yang

**Affiliations:** Guangdong Provincial Key Laboratory of Food, Nutrition and Health, Department of Nutrition, School of Public Health Sun Yat-sen University, Guangzhou, China

**Keywords:** NAFLD, klotho, cognitive performance, DSST, AFT

## Abstract

**Introduction:**

This study investigated the potential link between serum klotho levels and cognitive function in patients with non-alcoholic fatty liver disease (NAFLD).

**Materials and Methods:**

Utilizing NHANES data from 2011 to 2014, the research included 356 eligible participants. NAFLD was identified with the United States Fatty Liver Index (US-FLI), and cognition was measured by various tests including the Animal Fluency Test (AFT), Digit Symbol Substitution Test (DSST), Immediate Recall Test (IRT), and Delayed Recall Test (DRT). Weighted logistic regression and restricted cubic splines were employed to analyze the relationship between klotho levels and cognitive scores.

**Results:**

A significant nonlinear association was observed between klotho levels and the performance in DSST and Delayed Recall Test (DRT). After controlling for confounding factors, the study found a positive association between higher serum klotho levels and improved cognitive performance in both AFT and DSST. However, there was no significant relationship between klotho levels and the IRT or DRT, regardless of whether the natural logarithm or quartile was considered.

**Discussion:**

The findings suggest that a higher serum klotho level may be positively correlated with better cognitive performance in NAFLD patients.

## 1 Introduction

Nonalcoholic fatty liver disease (NAFLD) has a high prevalence worldwide, and there is evidence suggesting that NAFLD is associated with an increased risk of dementia and cognitive decline (Weinstein et al., 2019; Kim et al., 2022). Since the discovery of klotho protein at the end of the last century, many animal studies have shown that klotho can effectively delay aging and protect cognitive function (Kuro-o, 2009). A study involving 10,949 American adults found a link negative association between NAFLD and klotho levels (Chi et al., 2023). However, the relationship between cognitive function and klotho levels in patients with NAFLD requires further exploration.

The definition of NAFLD include evidence of hepatic steatosis (HS), as well as the absence of significant alcohol consumption, and other known causes of hepatic fat accumulation (Rinella et al., 2024). Patients with NAFLD may progress to nonalcoholic steatohepatitis (NASH), leading to the development of liver fibrosis and eventually liver cancer (Thomas et al., 2024). The main feature of NAFLD is the excessive accumulation of triglycerides in liver cells. However, the reasons for the ongoing deterioration of NAFLD are still unclear (Engin, 2017). The prevalence of NAFLD in the general population of the United States was 26% according to a 2016 survey (Younossi et al., 2016), and there is a global trend of increasing. It would be a huge burden on society (Golabi et al., 2024; Riazi et al., 2022).

NAFLD is a systemic disease closely linked to various comorbidities, including cardiovascular disease, chronic kidney disease, and several types of cancer (Duell et al., 2022; Thomas et al., 2024; Marcuccilli and Chonchol, 2016). The result that NAFLD patients has smaller total cerebral brain volume suggests a possible association between NAFLD and brain aging (Weinstein et al., 2018). NAFLD may be a risk factor for central nervous system dysfunction. The reduced peripheral clearance capacity in NAFLD patients may lead to the inability to excrete substances such as amyloid-beta peptide (Aβ), thereby resulting in brain damage (Estrada et al., 2019). A study was conducted on 4,472 adults aged 20–59 found that NAFLD was independently associated with lower cognitive performance (Seo et al., 2016).

Since the discovery of the klotho protein in 1997, it has been shown to play a crucial role in aging (Abraham and Li, 2022; Kuro et al., 1997). Currently, three types of klotho have been identified, including full-length transmembrane klotho (m-klotho), soluble klotho (s-klotho), and secreted klotho (Xu and Sun, 2015). The klotho gene is primarily expressed in the kidneys and choroid plexus of the brain (Wang and Sun, 2009, 2010). Its levels in the body gradually decrease with age. Many experiments have been conducted in animal studies, mice lacking klotho exhibit manifestations resembling premature aging that manifest universally. These mice with restricted klotho expression stop growing after 3–4 weeks of life and die prematurely at 8–9 weeks (Kuro et al., 1997). Additionally, these mice experienced rapid atrophy of the thymus, thinning of the skin, and progressive emphysema around the lungs—symptoms similar to natural aging rather than pathological changes (Kawaguchi et al., 1999; Kuro et al., 1997). Notably, these Klotho-deficient mice also exhibited signs of impaired cognitive function in new object recognition and conditioned fear tests, demonstrating deficits in visual recognition memory and associative fear memory (Nagai et al., 2003). It had been found that klotho had a protective effect on the cognitive function of animals through many ways, such as overexpression of klotho protein improved the clearance of amyloid beta in Alzheimer’s mice (Zhao et al., 2020). And a study found that klotho increased Forkhead box O3a (FOXO-3a) activity and catalase levels in mouse brain astrocytes, as well as increase proteasome activity in neurons, thereby regulating brain energy metabolism and redox state (Orellana et al., 2023). The levels of klotho in the human body are associated with various diseases such as cardiovascular disease, renal fibrosis, and malignant tumors (Liu and Chen, 2023; Pei et al., 2023; Qiao et al., 2023). One study found lower levels of klotho in the cerebrospinal fluid of Alzheimer’s disease (AD) patients (Semba et al., 2014), suggesting a potential link between lower klotho levels and cognitive function (Linghui et al., 2023).

Research on the association between NAFLD and klotho is still scarce. A study based on NHANES data from 2007 to 2016 found that lower levels of *α*-Klotho protein in the blood were associated with NAFLD, particularly in individuals under 51 years of age, females, and non-Hispanic white populations. The study also suggested that increased levels of α-Klotho might have potential benefits for the treatment of NAFLD (Chi et al., 2023).

To date, the relationship between the levels of serum klotho and cognitive function among patients with NAFLD remains unclear. Thus, we aimed to investigate the association between levels of serum klotho and cognitive function among individuals with NAFLD.

## 2 Materials and methods

### 2.1 Study design and participants

This study utilized publicly available data from the National Health and Nutrition Examination Survey (NHANES), accessible at https://www.cdc.gov/nchs/nhanes/index.htm. Data from two consecutive NHANES cycles, 2011–2014, were pooled for this study. Included participants were over 60 years of age and meet the criteria for NAFLD; individuals with missing data on serum klotho or cognitive function were excluded.

**Figure 1:**
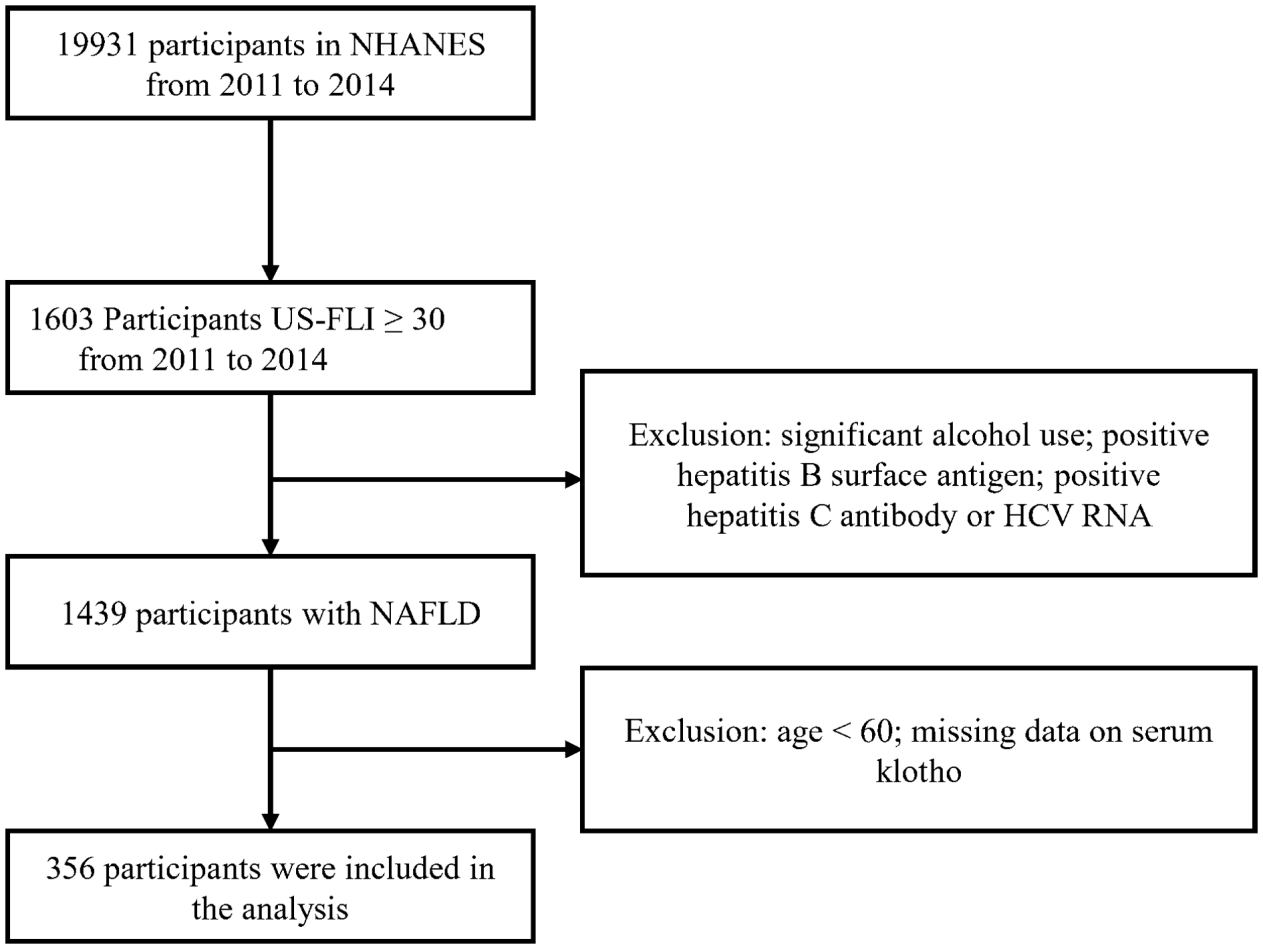
Flow chart of participants’ enrollment.

According to the selection criteria, we selected 356 participants from 19,931 participants. The screening process is as follows: first, 1,603 participants were selected based on the criterion of US-FLI ≥ 30 from the total of 19,931 participants. Then, individuals with significant alcohol consumption, as well as those testing positive for hepatitis B surface antigen, positive for hepatitis C antibody, or HCV RNA were excluded. Lastly, participants below the age of 60 and those with missing klotho data were removed, resulting in the inclusion of 356 participants who met the criteria (Figure 1).

### 2.2 Definition of NAFLD

The gold standard for diagnosis of nonalcoholic fatty liver disease is liver biopsy. Due to the invasive nature of liver biopsy and possible complications, non-invasive testing (NIT) is increasingly acknowledged in clinical practice. Many models have been proposed. A widely used model modified on the basis of U.S. population data is the United States Fatty Liver Index (US-FLI), whose the area under the receiver operating characteristic (ROC) curve [AUC; 95% confidence interval (CI)] was 0.80 (0.77–0.83) (Ruhl and Everhart, 2015). US-FLI was calculated based on race, age, waist circumference, blood glucose, and other indicators. NAFLD is defined when the US-FLI value is ≥30 and the participant has no other established risk factors for chronic liver diseases (Ruhl and Everhart, 2015; Sourianarayanane and McCullough, 2022) including viral hepatitis and heavy alcohol intake (≥2 drinks per day for men or ≥ 1 drink per day for women).

### 2.3 Measurement of cognitive performance

To assess cognitive performance in participants older than 60 years of age in the 2011–2014 NHANES survey. The interviews were conducted by trained interviewers in the Mobile Examination Center (MEC Interview) and scored after the interviews were completed. A lower score indicated poorer cognitive function, and there was no defined threshold for scoring. The Consortium to Establish a Registry for Alzheimer’s disease word list learning subtest (CERAD W-L), the Animal Fluency Test (AFT) and the Digit Symbol Substitution Test (DSST) were used in the cognitive performance test. The Immediate Recall Test (IRT) and one Delayed Recall Test (DRT) of the CERAD W-L test were used in cognitive performance evaluation to assess immediate and delayed learning abilities for new verbal information (memory domain), with a total score of 10 (Morris et al., 1989). This test has been utilized in many epidemiological studies (Fillenbaum et al., 2008; Gao et al., 2009). The AFT assesses verbal fluency by asking participants to name as many animals as possible within 1 min, earning one point for each animal named, which assesses executive function. The test scores have been shown to differentiate individuals with mild cognitive impairment from those with more severe cognitive impairment (such as Alzheimer’s disease) (Fillenbaum et al., 2008; Gao et al., 2009). DSST is a test module of the Wechsler Adult Intelligence Scale (WAIS III). The exercise is conducted using a paper form that has a key at the top containing 9 numbers paired with symbols. Participants had 2 min to copy the corresponding symbols in the 133 boxes that adjoin the numbers. It relies on processing speed, sustained attention, and working memory (Tulsky et al., 2001). Detailed questionnaires and scores are available on the NHANES website.

### 2.4 Determination of serum klotho levels

Serum samples collected during the 2011–2014 cycles of NHANES were received on dry ice and stored at-80°C in The Northwest Lipid Metabolism and Diabetes Research Laboratories, Division of Metabolism, Endocrinology, and Nutrition, University of Washington determined klotho concentrations in these samples using an ELISA kit from IBL International, Japan. The klotho concentration in each sample was measured twice in a series, and the final value was the average of the two measurements. If the difference between the repeated measurements exceeded 10 percent, the klotho concentration was re-measured. And if the value of a quality control sample was not within the 2SD of the assigned value, the entire analytical run was rejected, and sample analyses repeated. The sensitivity of the test was 6 pg/mL. More details are available on the NHANES website.

### 2.5 Covariates

The following variables were included in this study, such as age (≤69, 70–79), sex (male and female), race (Mexican American, Non-Hispanic white, Non-Hispanic black, other), education (< high school, high school, > high school), the family income–poverty ratio (FMPIR) calculated as the ratio of household income to the poverty line (less than 1.0, between 1.0 and 3.0, and greater than 3.0), smoking status (never smoker, former smoker, current smoker), body mass index (BMI). Hypertension was defined as a systolic blood pressure of more than 140 mm Hg or a diastolic blood pressure of more than 90 mm Hg or having been explicitly told by a doctor to have hypertension or were taking high blood pressure medication (Beckman and Members, 2023). Depression was defined as participants who scored 10 or above on the NHANES project’s Mental Health-Depression Screener (DPQ_G) (Kroenke et al., 2001). Estimated glomerular filtration rate (eGFR) was calculated based on the participants’ creatinine, age, sex, and race information (Levey et al., 2009). Antipsychotics can also affect participants’ cognitive status, so we also included antipsychotics as a covariate, based on whether participants were taking antipsychotics such as Amitriptyline, Bupropion, and Citalopram. Questionnaire findings (self-reported physician diagnosis of high cholesterol, diabetes, stroke), laboratory data alanine transaminase (ALT), aspartate aminotransferase (AST), total cholesterol (TC), triglyceride (TG), low-density lipoprotein cholesterol (LDL-C), high-density lipoprotein cholesterol (HDL-C).

### 2.6 Statistical analysis

In this study, all analyses considered the complex sampling design of the NHANES. Data were presented as unweighted frequencies (weighted percentages) for categorical variables and as medians (interquartile range) for continuous variables. Differences among groups were compared using the Kruskal-Wallis test for continuous variables with non-normal distribution and the χ^2^ test with the Rao and Scott second-order correction for categorical variables. Multivariable linear regression models were used to assess the association between cognitive test scores and levels of serum klotho. We constructed two models for analysis: the first was crude model. In model 2, we adjusted for age, sex, race, education level, family income-poverty ratio, smoking status, BMI, stroke, diabetes, hypertension, depression, antipsychotic, eGFR, and TG. Restricted cubic spline analysis was used to examine the nonlinear relationship between klotho protein levels and cognitive test scores. A multivariate linear regression model was used for subgroup analysis included age, sex, education level, diabetes mellitus and other variables to analyze the relationship between cognitive function score and klotho. Covariates with missing values were imputed using the multiple imputation by chained equations method R software (version 4.2.2) was used for statistical analysis in the study, and the threshold for statistical significance was set at *p* < 0.05.

## 3 Results

### 3.1 Baseline characteristics

A total of 356 participants were included in this study. The baseline characteristics of 356 participants with NAFLD according to quartile of serum klotho were summarized in Table 1. Similarities were found in the percentages of sex, race and ethnicity, education levels, family income–poverty ratio, smoking status, hypertension, depression, antipsychotic medication, high cholesterol, stroke, and the levels of ALT, AST, TC, LDL-C, HDL-Cand the score of IRT, DRT, AFT among four groups (all *p* > 0.05). Statistical significances were found in age, diabetes, TG, eGFR and DSST. The score of DSST increased from group Q1 to Q3 and decreased in group Q4 (*p* < 0.05). Age and the number of people with diabetes decreased as the klotho rating (quartile) increased (Table 1).

**Table 1:** Characteristics of participants with NAFLD according to quartiles of serum klotho.

Characteristic	Overall (*N* = 356)	Serum klotho, pg/mL				
		Q1 (*N* = 91)	Q2 (*N* = 87)	Q3 (*N* = 89)	Q4 (*N* = 89)	*p*
Age, years	66.0 (63.0, 71.0)	67.0 (64.3, 71.0)	67.1 (62.7, 72.8)	65.0 (63.0, 69.7)	65.8 (62.0, 72.0)	0.031
Age (years), *n* (%)						0.371
≤ 69	212 (65.7)	53 (63.9)	50 (56.8)	56 (74.6)	53 (65.9)	
70–79	144 (34.3)	38 (36.1)	37 (43.2)	33 (25.4)	36 (34.1)	
Gender, *n* (%)						0.981
Male	186 (50.6)	45 (48.3)	53 (50.7)	45 (51.6)	43 (51.8)	
Female	170 (49.4)	46 (51.7)	34 (49.3)	44 (48.4)	46 (48.2)	
Race, *n* (%)						0.977
Mexican American	60 (6.3)	15 (5.9)	16 (7.1)	14 (5.7)	15 (6.6)	
Non-Hispanic White	175 (79.6)	48 (78.3)	39 (78.1)	46 (82.5)	42 (79.4)	
Non-Hispanic Black	43 (4.3)	13 (5.3)	8 (3.8)	8 (3.2)	14 (4.6)	
Other	78 (9.8)	15 (10.4)	24 (11.1)	21 (8.7)	18 (9.4)	
Education, *n* (%)						0.439
< High school	110 (19.4)	26 (13.3)	23 (23.3)	31 (14.8)	30 (26.7)	
High school	87 (24.8)	27 (31.5)	16 (17.6)	23 (27.1)	21 (22.1)	
Some college or above	159 (55.7)	38 (55.2)	48 (59.1)	35 (58.0)	38 (51.2)	
FMPIR, *n* (%)						0.866
< 1.0	60 (8.5)	18 (9.7)	12 (8.5)	16 (8.8)	14 (7.2)	
1.0–3.0	146 (40.0)	37 (38.2)	35 (32.8)	39 (43.3)	35 (44.7)	
> 3.0	117 (51.4)	32 (52.2)	30 (58.7)	23 (47.9)	32 (48.2)	
Smoking status, *n* (%)						0.271
Never smoker	162 (43.6)	45 (46.0)	32 (41.8)	40 (46.1)	45 (40.4)	
Former smoker	159 (46.7)	37 (45.7)	51 (56.4)	37 (42.6)	34 (43.7)	
Current smoker	35 (9.7)	9 (8.3)	4 (1.8)	12 (11.3)	10 (15.9)	
BMI, kg/m^2^	33.1 (29.2, 38.0)	31.1 (27.7, 39.4)	33.6 (30.6, 38.1)	33.7 (29.5, 38.7)	31.2 (29.1, 35.1)	0.237
BMI (kg/m2), *n* (%)						0.169
< 30.0	109 (30.1)	28 (37.8)	28 (18.5)	26 (31.2)	27 (31.4)	
≥ 30.0	244 (69.9)	61 (62.2)	59 (81.5)	62 (68.8)	62 (68.6)	
Hypertension, *n* (%)						0.430
Yes	287.0 (81.1)	76.0 (88.2)	69.0 (77.0)	68.0 (76.0)	74.0 (82.8)	
No	69.0 (18.9)	15.0 (11.8)	18.0 (23.0)	21.0 (24.0)	15.0 (17.2)	
High cholesterol, *n* (%)						0.307
Yes	223 (67.4)	62 (73.9)	54 (72.7)	53 (57.5)	54 (66.3)	
No	128 (32.6)	29 (26.1)	32 (27.3)	34 (42.5)	33 (33.7)	
Diabetes, *n* (%)						0.009
Yes	122 (32.7)	39 (49.0)	30 (32.5)	28 (30.2)	25 (19.7)	
No	234 (67.3)	52 (51.0)	57 (67.5)	61 (69.8)	64 (80.3)	
Depression						0.578
Yes	42.0 (9.1)	12.0 (9.4)	10.0 (13.2)	12.0 (8.6)	8.0 (5.8)	
No	314.0 (90.9)	79.0 (90.6)	77.0 (86.8)	77.0 (91.4)	81.0 (94.2)	
Antipsychotic						0.243
Yes	22.0 (6.7)	6.0 (9.5)	7.0 (10.8)	5.0 (5.1)	4.0 (2.2)	
No	334.0 (93.3)	85.0 (90.5)	80.0 (89.2)	84.0 (94.9)	85.0 (97.8)	
Stroke, *n* (%)						0.747
Yes	24 (6.6)	7 (8.8)	4 (4.3)	8 (7.7)	5 (5.1)	
No	332 (93.4)	84 (91.2)	83 (95.7)	81 (92.3)	84 (94.9)	
ALT, U/L	22.0 (18.0, 28.0)	21.0 (17.9, 27.0)	22.1 (20.0, 30.0)	22.0 (18.0, 27.8)	23.0 (18.4, 27.9)	0.314
AST, U/L	24.0 (20.0, 27.0)	22.0 (19.0, 26.0)	25.0 (21.0, 27.0)	23.0 (20.0, 26.0)	24.0 (21.0, 28.0)	0.175
TC, mg/dL	181.3 (155.0, 209.0)	177.6 (149.0, 203.0)	183.5 (156.7, 207.0)	187.0 (162.2, 207.8)	180.9 (154.5, 213.6)	0.716
TG, mg/dL	145.0 (101.0, 192.0)	157.7 (123.3, 215.6)	138.1 (97.0, 210.9)	153.2 (122.6, 189.8)	121.1 (91.0, 164.4)	0.008
LDL-C, mg/dL	106.0 (78.0, 129.0)	98.4 (76.0, 121.0)	103.5 (73.5, 131.0)	109.5 (91.0, 132.6)	106.5 (77.5, 134.9)	0.663
HDL-C, mg/dL	47.0 (40.0, 54.0)	46.0 (39.0, 53.0)	47.0 (41.0, 55.0)	45.0 (39.0, 53.0)	49.0 (44.8, 55.6)	0.118
eGFR, mL/min/1.73m^2^	76.6 (61.7, 88.9)	70.6 (50.8, 89.5)	75.7 (59.7, 83.8)	78.8 (66.3, 90.2)	76.6 (69.5, 89.4)	0.045
IRT	20.0 (17.0, 22.0)	20.0 (17.0, 23.0)	19.7 (17.0, 22.0)	20.0 (18.0, 22.0)	19.0 (17.0, 21.0)	0.711
DRT	6.0 (5.0, 8.0)	7.0 (5.0, 8.1)	6.0 (5.0, 9.0)	7.0 (5.0, 8.0)	6.0 (5.0, 8.0)	0.885
AFT	17.0 (14.0, 21.0)	16.0 (13.0, 19.3)	16.0 (14.6, 20.0)	18.0 (14.0, 22.0)	19.6 (14.0, 20.5)	0.071
DSST	53.0 (43.0, 61.7)	50.0 (40.0, 56.4)	53.0 (42.0, 63.0)	58.4 (47.7, 63.6)	50.0 (42.9, 57.6)	0.008

Q1, Q2, Q3, Q4 are first quartile, second quartile, third quartile and max quartile; FMPIR, family income–poverty ratio; BMI, Body Mass Index; ALT, Alanine aminotransferase; AST, Aspartate aminotransferase; TC, Total Cholesterol; TG, Triglyceride; LDL-C, Low-Density Lipoprotein Cholesterol; HDL-C, High-Density Lipoprotein Cholesterol; eGFR, estimated glomerular filtration rate; IRT, Immediate Recall Test; DRT, Delayed Recall Test; AFT, Animal Fluency Test; DSST, Digit Symbol Substitution Test.

### 3.2 Association between klotho and cognitive function in NAFLD

**Figure 2:**
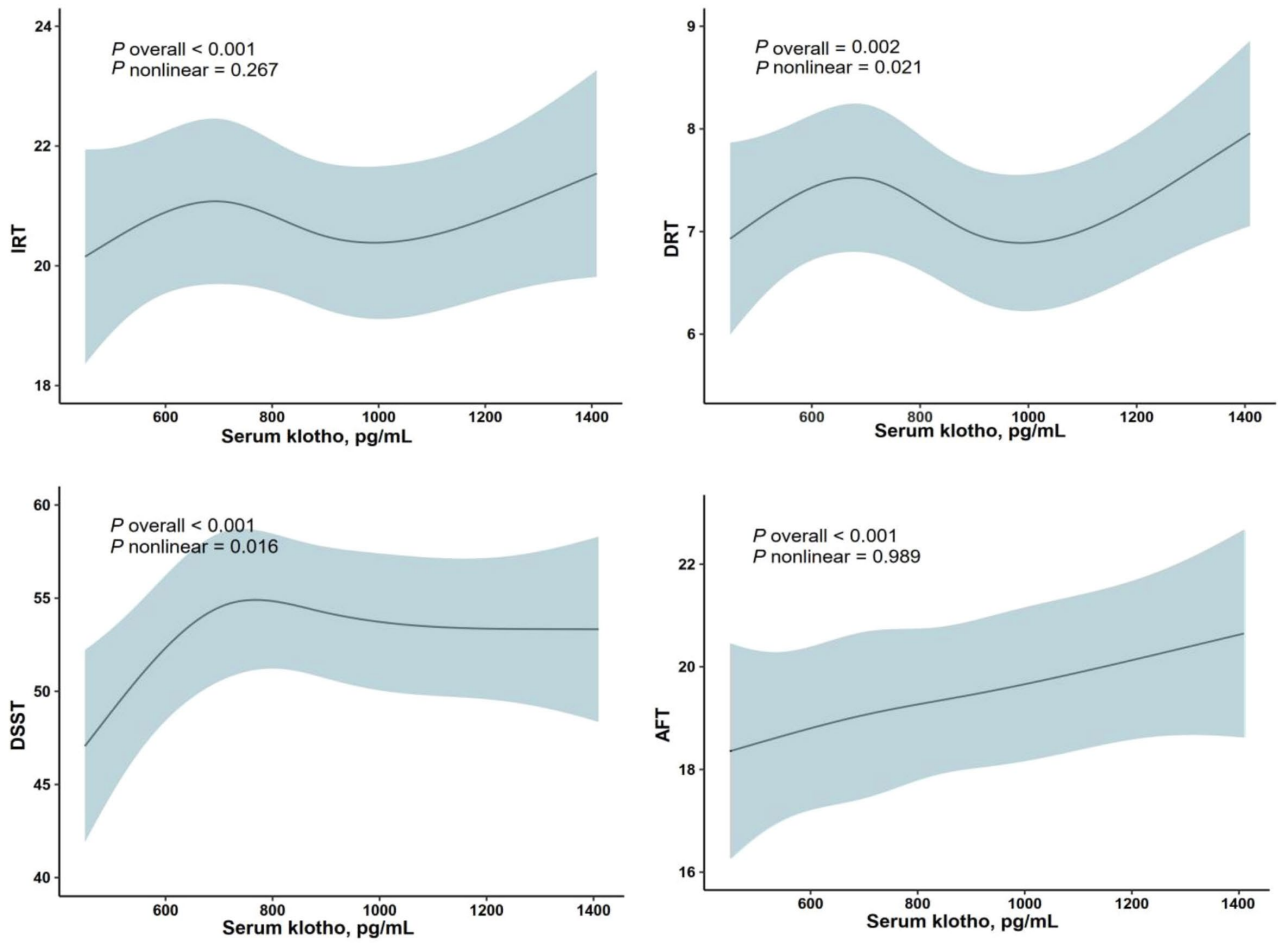
Restricted cubic spline (RCS) plot of the association between Klotho and cognitive function.

Nonlinear relationship was found between DSST and serum klotho (*p* nonlinear = 0.016) in the restricted cubic splines. Similarly, a nonlinear relationship between DRT and serum klotho was also observed (*p* nonlinear = 0.021). However, there was no significant nonlinear relationship between serum klotho and either IRT (*p* nonlinear = 0.267) or AFT (*p* nonlinear = 0.989; both *p* < 0.001 for overall; Figure 2).

### 3.3 Association between serum klotho and cognitive performance

There was no significant association between serum klotho and IRT or DRT, regardless of whether the natural logarithm or quartile was taken for klotho. The *β* value (95% CI) of klotho (natural logarithm) and DSST in Model 1 and Model 2 were 4.05 (0.25, 7.84) and 5.47 (1.60, 9.34), and *P* were 0.012 and 0.040, respectively. The β value (95% CI) values for klotho (natural logarithm) and AFT in Models 1 and 2 were 1.70 (0.41, 2.98) and 2.03 (0.11, 3.96), and *p* were 0.012 and 0.040. In the analysis of the association between klotho actual values and cognitive scores, the second and third quartiles (Q2 and Q3) exhibited a β (95% CI) of 4.99 (0.64, 9.34) and 6 (3.06, 8.94) in comparison to the first quartile (Q1) for the DSST at model 1. In comparison to the reference group (Q1), the *β* value (95% CI) of AFT in Model 1 for Q4 in klotho (quartile) was 1.52 (0.05, 3.00; Table 2).

**Table 2:** Association between serum klotho and cognitive performance.

	Model	Klotho (natural logarithm)	P	Klotho (quartile), pg/mL			
				Q1 (≤ 656.6)	Q2 (656.7–801.3)	Q3 (801.4–983.7)	Q4 (> 983.7)
IRT	Model 1	0.34 (−1.80, 2.48)	0.748	Ref	0.04 (−1.49, 1.56)	0.02 (−1.81, 1.84)	−0.38 (−2.18, 1.41)
	Model 2	0.74 (−1.49, 2.98)	0.482	Ref	0.1 (−1.46, 1.65)	−0.14 (−2.2, 1.91)	−0.16 (−2.29, 1.98)
DRT	Model 1	0.11 (−1.34, 1.57)	0.873	Ref	−0.09 (−1.00, 0.81)	−0.11 (−1.20, 0.98)	−0.22 (−1.29, 0.85)
	Model 2	0.38 (−1.09, 1.85)	0.581	Ref	−0.16 (−0.96, 0.63)	−0.18 (−1.2, 0.83)	−0.08 (−1.24, 1.08)
AFT	Model 1	1.70 (0.41, 2.98)	**0.012**	Ref	0.61 (−1.33, 2.55)	1.92 (−0.01, 3.84)	1.16 (−0.05, 2.38)
	Model 2	2.03 (0.11, 3.96)	**0.040**	Ref	0.79 (−0.81, 2.38)	1.71 (−0.1, 3.52)	**1.52 (0.05, 3.00)**
DSST	Model 1	4.05 (0.25, 7.84)	**0.037**	Ref	5.32 (−0.02, 10.65)	**7.55 (2.76, 12.33)**	2.24 (−1.07, 5.56)
	Model 2	5.47 (1.6, 9.34)	**0.009**	Ref	**4.99 (0.64, 9.34)**	**6 (3.06, 8.94)**	2.89 (−0.65, 6.42)

Model 1: crude model. Model 2: adjust for age, gender, race, education levels, family income–poverty ratio, smoking status, BMI, Stroke, hypertension, antipsychotic, Depression, eGFR, Diabetes, and TG. Bold text indicates a *p*-value < 0.05, indicating a statistical difference between the two.

### 3.4 Subgroup analysis: associations of serum klotho with DSST score

We found a statistical significance between klotho (natural logarithm) and DSST when controlling for age (*p* = 0.029 for interaction). Compared with the reference group (first quartile), the DSST score β value in the third quartile for the subgroup with FMPIR >3.0 was 10.36 (95% CI: 1.96, 18.77), the DSST score *β* value of the fourth quartile was 9.43 (95% CI: 2.29, 16.56). In addition, we also analyzed the associations of serum klotho with scores of IRT, DRT, AFT in various subgroups. In Supplementary Table S3, we found a statistical significance between klotho (natural logarithm) and AFT when controlling diabetes (*p* = 0.004) (Table 3; Supplementary Tables S1–S3).

**Table 3:** Associations of serum klotho with DSST score in various subgroups.

Characteristic	Serum Klotho, pg/mL				
	Q1 (≤656.6)	Q2 (656.7–801.3)	Q3 (801.4–983.7)	Q4 (≥ 983.8)	P _interaction_
Age, years					
< 70	Ref	**10.44 (3.4, 17.49)**	**8.6 (4.47, 12.73)**	5.42 (−0.77, 11.62)	0.076
≥ 70	Ref	0.67 (−6.06, 7.4)	3.27 (−4.45, 10.99)	0.94 (−9.01, 10.89)	
Gender					
Male	Ref	5.35 (−0.42, 11.12)	5.5 (−0.14, 11.13)	1.24 (−3.94, 6.43)	0.819
Female	Ref	4.86 (−0.7, 10.42)	**7.22 (2.32, 12.12)**	5.21 (−1.78, 12.2)	
Education					
≤ High school	Ref	**7.75 (0.9, 14.6)**	**7.26 (1.4, 13.13)**	4.17 (−1.18, 9.52)	0.436
Some college or above	Ref	3.19 (−2.94, 9.31)	**4.88 (0.7, 9.05)**	−0.41 (−6.93, 6.12)	
FMPIR					
≤ 3.0	Ref	1.34 (−5.42, 8.1)	3.15 (−1.66, 7.96)	2.56 (−2.12, 7.24)	**0.029**
> 3.0	Ref	**10.36 (1.96, 18.77)**	**9.43 (2.29, 16.56)**	5.37 (−5.49, 16.24)	
Smoking status					
Never	Ref	4.5 (−5.58, 14.57)	6.45 (−1.01, 13.92)	6.9 (−0.78, 14.59)	0.368
Former or current	Ref	**7.24 (1.22, 13.27)**	**5.67 (1.19, 10.14)**	1.2 (−2.53, 4.92)	
BMI					
< 30.0	Ref	−0.85 (−9.58, 7.89)	4.99 (−3.61, 13.59)	−4.67 (−12.73, 3.4)	0.508
≥ 30.0	Ref	5.91 (−0.12, 11.94)	**5.85 (0.72, 10.97)**	4.76 (−0.72, 10.25)	
Diabetes					
Yes	Ref	2.38 (−7.19, 11.95)	6.75 (−1.55, 15.04)	2.94 (−5.64, 11.51)	0.371
No	Ref	**7.52 (2.28, 12.77)**	**6.33 (1.47, 11.18)**	2.91 (−2.72, 8.54)	

NAFLD, Nonalcoholic fatty liver disease; BMI, body mass index; NHANES, National Health and Nutrition Examination Survey. Data are presented as β value (95% CI). Adjust for age, gender, race, education levels, family income–poverty ratio, smoking status, BMI, Stroke, hypertension, antipsychotic, Depression, eGFR, Diabetes, and TG. The strata variable was not included when stratifying by itself. Bold text indicates a *p*-value < 0.05, indicating a statistical difference between the two.

## 4 Discussion

To our knowledge, this study was the first study to examine the association between the levels of serum klotho and cognitive function (the scores of IRT, DRT, AFT, and DSST) among individuals with NAFLD. In our analysis, no statistical significances were observed between IRT and DRT scores and serum klotho levels; However, AFT scores exhibited a positive association with serum klotho levels, while DSST scores may demonstrate a non-linear relationship with serum klotho levels.

The klotho protein has long been recognized for its potential to extend lifespan and protect various organs (Kuro et al., 1997), Numerous studies have demonstrated its neuroprotective effects on the brain and nervous system, suggesting a potential role in preventing cognitive impairment.” Co-incubation of klotho with glia conditioned medium + lipopolysaccharides has shown complete restoration of low-concentration glia conditioned medium—lipopolysaccharides induced neuronal toxicity (Nakao et al., 2022). Mice with klotho deficiency exhibit immature hippocampal neurons, while overexpression of klotho in the hippocampal region leads to an increase in neuron count and influences hippocampus-dependent spatial memory function (Laszczyk et al., 2017).

In previous studies, no association was found between IRT, DRT and serum klotho levels (Linghui et al., 2023; Ge et al., 2024). In our study, no statistically significant results were found, consistent with the results of previous studies. IRT and DRT are tests of the ability of short-term memory. In many animal experiments, it has been found that the expression or supplementation of klotho will improve memory, injection of a lentiviral vector capable of delivering and maintaining klotho expression in seven-month-old mice led to a significant increase in klotho expression in the brains of mice after 3 months of feeding, and the treatment reduced memory impairment and neuronal loss (Zhou et al., 2018). In another study, low doses of rhesus klotho protein administered subcutaneously to mice and older rhesus monkeys enhanced memory function, but no lasting cognitive effects were observed in rhesus monkeys similar to the high doses of klotho protein in mice, this may be due to the more complex brain structure of primates (Castner et al., 2023). The study did not find a significant association between klotho levels and human memory performance, possibly due to the consistent maintenance of a certain level of klotho in humans. The specific mechanism needs to be further studied.

In the analysis between individual factors and klotho, DSST obtained statistically significant results in the comparison with klotho. It can be seen from our results that the DSST score increases with the increase of serum klotho, but the DSST score does not continue to increase at the fourth quartile level of the serum klotho, instead of a downward trend. This nonlinear relationship has been less frequently reported in previous similar studies. A comparable association was observed in a study on the association between serum klotho concentration and cognitive ability in elderly patients with nephropathy and proteinuria, which also demonstrated an initial increase followed by a subsequent decrease (Zhang and Zhang, 2023). However, the decline in the later stage was more pronounced than that observed in this study. These findings suggest a strong relationship between lower klotho levels and lower cognitive performance in the NAFLD population.

The levels of klotho are closely associated with stress responses which plays a role in reducing inflammation. A decrease in klotho concentration is linked to an increase in oxidative stress response (Lin and Beal, 2006; Kuro-o, 2009). Research data suggested a strong association between klotho and depression, which may be attributed to oxidative stress (Gold et al., 2013). Additionally, literature also suggested a relationship between klotho, cognitive function, and stress response (Gao et al., 2021). In the “double-hit” hypothesis of NAFLD, inflammation mediated by oxidative stress is considered as the “second hit” (Chitturi and Farrell, 2001). Further investigation is needed to explore whether stress response may also play a significant role in the association between klotho levels and cognitive function in NAFLD.

In our subgroup analysis, when controlling for diabetes variables, we identified a statistical significance between klotho and AFT scores (*p* = 0.004). Compared with the reference group (first quartile), a statistical significance was observed in AFT scores within the third quartile of the diabetes subgroup, with a *β* value of 4.74 (95% CI: 1.12, 8.35; Supplementary Table S3). Previous studies on diabetes have shown similar results, indicating a positive correlation between the levels of klotho and cognition in patients with diabetes (Zhang et al., 2023). Some statistically significant results in the diabetes variable were also found in the subgroup analysis between DSST scores and klotho, but in people without diabetes (shown in Table 3). And these results suggest that having lower klotho levels or klotho proteins may have a greater impact on cognitive function in patients with comorbidities.

It should be noted that this study has certain limitations. First, it is a cross-sectional study, which precludes any causal inferences. Second, the sample size of study participants who were screened and ultimately included in the analysis was relatively small. Third, as the cognitive score test was only administered to participants over the age of 60, caution is required when attempting to generalize the findings to other age groups with NAFLD.

In the future, more in-depth studies should be conducted to explore the association in the NAFLD patient population, including prospective studies across different countries, races, regions, and a wider age group, to further verify the predictive value of klotho levels in the cognitive function of NAFLD patients.

## 5 Conclusion

In conclusion, a statistical significance exists between klotho and the scores of AFT and DSST, indicating a higher serum klotho level may be positively correlated with better cognitive performance in NAFLD patients. Our study suggests that routine testing of serum klotho can be considered in NAFLD patients for early detection of cognitive decline.

## Data Availability

The original contributions presented in the study are included in the article/Supplementary material, further inquiries can be directed to the corresponding author/s.

## References

[ref1] Abraham C. R. Li A. (2022). Aging-suppressor klotho: prospects in diagnostics and therapeutics. Ageing Res. Rev. 82:101766. doi: 10.1016/j.arr.2022.101766, PMID: 36283617

[ref2] Beckman J. A. Members W. C. (2023). *2022 ACC/AHA guideline for the diagnosis and Management of Aortic Disease*. A Report of the American Heart Association/American College of Cardiology Joint Committee on Clinical Practice Guidelines. 10.1016/j.jtcvs.2023.04.023 PMC10784847 37389507

[ref3] Castner S. A. Gupta S. Wang D. Moreno A. J. Park C. Chen C.. (2023). Longevity factor klotho enhances cognition in aged nonhuman primates. Nat Aging 3, 931–937. doi: 10.1038/s43587-023-00441-x, PMID: 37400721 PMC10432271

[ref4] Chi Z. Teng Y. Liu Y. Gao L. Yang J. Zhang Z. (2023). Association between klotho and non-alcoholic fatty liver disease and liver fibrosis based on the NHANES 2007-2016. Ann. Hepatol. 28:101125. doi: 10.1016/j.aohep.2023.101125, PMID: 37286168

[ref5] Chitturi S. Farrell G. C. (2001). Etiopathogenesis of nonalcoholic steatohepatitis. Semin. Liver Dis. 21, 027–042. doi: 10.1055/s-2001-12927 11296694

[ref6] Duell P. B. Welty F. K. Miller M. Chait A. Hammond G. Ahmad Z.. (2022). Nonalcoholic fatty liver disease and cardiovascular risk: a scientific statement from the American Heart Association. Arterioscler. Thromb. Vasc. Biol. 42, e168–e185. doi: 10.1161/ATV.0000000000000153, PMID: 35418240

[ref7] Engin A. (2017). “Non-alcoholic fatty liver disease” in obesity and lipotoxicity. eds. Engin A. B. Engin A. (Cham: Springer International Publishing), 443–467.

[ref8] Estrada L. D. Ahumada P. Cabrera D. Arab J. P. (2019). Liver dysfunction as a novel player in Alzheimer’s progression: looking outside the brain. Front. Aging Neurosci. 11:174. doi: 10.3389/fnagi.2019.00174, PMID: 31379558 PMC6650779

[ref9] Fillenbaum G. G. van Belle G. Morris J. C. Mohs R. C. Mirra S. S. Davis P. C.. (2008). Consortium to establish a registry for Alzheimer’s disease (CERAD): the first twenty years. Alzheimers Dement. 4, 96–109. doi: 10.1016/j.jalz.2007.08.005, PMID: 18631955 PMC2808763

[ref10] Gao S. Jin Y. Unverzagt F. W. Liang C. Hall K. S. Ma F.. (2009). Hypertension and cognitive decline in rural elderly Chinese. J. Am. Geriatr. Soc. 57, 1051–1057. doi: 10.1111/j.1532-5415.2009.02267.x, PMID: 19507297 PMC2849159

[ref11] Gao X. Li Y. Sun Z. Xu H. Ma G. Deng Q.. (2021). Could α-klotho unlock the key between depression and dementia in the elderly: from animal to human studies. Mol. Neurobiol. 58, 2874–2885. doi: 10.1007/s12035-021-02313-0, PMID: 33527303

[ref12] Ge S. Dong F. Tian C. Yang C.-H. Liu M. Wei J. (2024). Serum soluble alpha-klotho klotho and cognitive functioning in older adults aged 60 and 79: an analysis of cross-sectional data of the National Health and nutrition examination survey 2011 to 2014. BMC Geriatr. 24:245. doi: 10.1186/s12877-024-04661-7, PMID: 38468203 PMC10929106

[ref13] Golabi P. Owrangi S. Younossi Z. M. (2024). Global perspective on nonalcoholic fatty liver disease and nonalcoholic steatohepatitis—prevalence, clinical impact, economic implications and management strategies. Aliment. Pharmacol. Ther. 59 Suppl 1, S1–S9. doi: 10.1111/apt.17833, PMID: 38813821

[ref14] Gold P. W. Licinio J. Pavlatou M. G. (2013). Pathological parainflammation and endoplasmic reticulum stress in depression: potential translational targets through the CNS insulin, klotho and PPAR-γ systems. Mol. Psychiatry 18, 154–165. doi: 10.1038/mp.2012.167, PMID: 23183489 PMC10064987

[ref15] Kawaguchi H. Manabe N. Miyaura C. Chikuda H. Nakamura K. Kuro-o M. (1999). Independent impairment of osteoblast and osteoclast differentiation in klotho mouse exhibiting low-turnover osteopenia. J. Clin. Invest. 104, 229–237. doi: 10.1172/JCI5705, PMID: 10430604 PMC408412

[ref16] Kim G. Oh C. H. Kim J. W. Jeong S. J. Oh I. Lee J. S.. (2022). Association between non-alcoholic fatty liver disease and the risk of dementia: a nationwide cohort study. Liver Int. 42, 1027–1036. doi: 10.1111/liv.15244 35289469

[ref17] Kroenke K. Spitzer R. L. Williams J. B. W. (2001). The PHQ-9. J. Gen. Intern. Med. 16, 606–613. doi: 10.1046/j.1525-1497.2001.016009606.x, PMID: 11556941 PMC1495268

[ref18] Kuro M. Matsumura Y. Aizawa H. Kawaguchi H. Suga T. Utsugi T.. (1997). Mutation of the mouse klotho gene leads to a syndrome resembling ageing. Nature 390, 45–51. 9363890 10.1038/36285

[ref19] Kuro-o M. (2009). Klotho and aging. Biochim. Biophys. Acta 1790, 1049–1058. doi: 10.1016/j.bbagen.2009.02.005, PMID: 19230844 PMC2743784

[ref20] Laszczyk A. M. Fox-Quick S. Vo H. T. Nettles D. Pugh P. C. Overstreet-Wadiche L.. (2017). Klotho regulates postnatal neurogenesis and protects against age-related spatial memory loss. Neurobiol. Aging 59, 41–54. doi: 10.1016/j.neurobiolaging.2017.07.008, PMID: 28837861 PMC5612914

[ref21] Levey A. S. Stevens L. A. Schmid C. H. Zhang Y. Castro A. F. III Feldman H. I.. (2009). A new equation to estimate glomerular filtration rate. Ann. Intern. Med. 150, 604–612. doi: 10.7326/0003-4819-150-9-200905050-00006, PMID: 19414839 PMC2763564

[ref22] Lin M. T. Beal M. F. (2006). Mitochondrial dysfunction and oxidative stress in neurodegenerative diseases. Nature 443, 787–795. doi: 10.1038/nature05292 17051205

[ref23] Linghui D. Simin Y. Zilong Z. Yuxiao L. Shi Q. Birong D. (2023). The relationship between serum klotho and cognitive performance in a nationally representative sample of US adults. Front. Aging Neurosci. 15:1053390. doi: 10.3389/fnagi.2023.1053390, PMID: 36819720 PMC9932504

[ref24] Liu Y. Chen M. (2023). Emerging role of α-klotho in energy metabolism and cardiometabolic diseases. Diabetes Metab. Syndr. 17:102854. doi: 10.1016/j.dsx.2023.102854 37722166

[ref25] Marcuccilli M. Chonchol M. (2016). NAFLD and chronic kidney disease. Int. J. Mol. Sci. 17:562. doi: 10.3390/ijms17040562, PMID: 27089331 PMC4849018

[ref26] Morris J. C. Heyman A. Mohs R. C. Hughes J. P. van Belle G. Fillenbaum G.. (1989). The consortium to establish a registry for Alzheimer’s disease (CERAD). Part I. Clinical and neuropsychological assessment of Alzheimer’s disease. Neurology 39, 1159–1165. doi: 10.1212/wnl.39.9.1159, PMID: 2771064

[ref27] Nagai T. Yamada K. Kim H.-C. Kim Y.-S. Noda Y. Imura A.. (2003). Cognition impairment in the genetic model of aging klotho gene mutant mice: a role of oxidative stress. FASEB J. 17, 50–52. doi: 10.1096/fj.02-0448fje, PMID: 12475907

[ref28] Nakao V. W. CHY M. de Sá Lima L. de Mello P. S. de Souza Port's N. M. Kinoshita P. F.. (2022). Neuroprotective action of α-Klotho against LPS-activated glia conditioned medium in primary neuronal culture. Sci. Rep. 12:18884. doi: 10.1038/s41598-022-21132-4, PMID: 36344527 PMC9640694

[ref29] Orellana A. M. Mazucanti C. H. Dos Anjos L. P. de Sá Lima L. Kawamoto E. M. Scavone C. (2023). Klotho increases antioxidant defenses in astrocytes and ubiquitin-proteasome activity in neurons. Sci. Rep. 13:15080. doi: 10.1038/s41598-023-41166-6, PMID: 37699938 PMC10497516

[ref30] Pei Y. Miu M. Mao X. Chen W. Zhu J. (2023). α-Klotho: an early risk-predictive biomarker for acute kidney injury in patients with acute myocardial infarction. Int. J. Clin. Pract. 2023, 8244545–8244548. doi: 10.1155/2023/8244545, PMID: 38187354 PMC10771924

[ref31] Qiao Y. Liu F. Peng Y. Wang P. Ma B. Li L.. (2023). Association of serum klotho levels with cancer and cancer mortality: Evidence from National Health and nutrition examination survey. Cancer Med. 12, 1922–1934. doi: 10.1002/cam4.5027, PMID: 35841322 PMC9883546

[ref32] Riazi K. Azhari H. Charette J. H. Underwood F. E. King J. A. Afshar E. E.. (2022). The prevalence and incidence of NAFLD worldwide: a systematic review and meta-analysis. Lancet Gastroenterol. Hepatol. 7, 851–861. doi: 10.1016/S2468-1253(22)00165-0, PMID: 35798021

[ref33] Rinella M. E. Lazarus J. V. Ratziu V. Francque S. M. Sanyal A. J. Kanwal F.. (2024). A multisociety Delphi consensus statement on new fatty liver disease nomenclature. Ann. Hepatol. 29:101133. doi: 10.1016/j.aohep.2023.101133, PMID: 37364816

[ref34] Ruhl C. E. Everhart J. E. (2015). Fatty liver indices in the multiethnic United States National Health and nutrition examination survey. Aliment. Pharmacol. Ther. 41, 65–76. doi: 10.1111/apt.13012, PMID: 25376360

[ref35] Semba R. D. Moghekar A. R. Hu J. Sun K. Turner R. Ferrucci L.. (2014). Klotho in the cerebrospinal fluid of adults with and without Alzheimer’s disease. Neurosci. Lett. 558, 37–40. doi: 10.1016/j.neulet.2013.10.058, PMID: 24211693 PMC4037850

[ref36] Seo S. W. Gottesman R. F. Clark J. M. Hernaez R. Chang Y. Kim C.. (2016). Nonalcoholic fatty liver disease is associated with cognitive function in adults. Neurology 86, 1136–1142. doi: 10.1212/WNL.0000000000002498, PMID: 26911638 PMC4820136

[ref37] Sourianarayanane A. McCullough A. J. (2022). Accuracy of steatosis and fibrosis NAFLD scores in relation to vibration controlled transient elastography: an NHANES analysis. Clin. Res. Hepatol. Gastroenterol. 46:101997. doi: 10.1016/j.clinre.2022.101997, PMID: 35842111

[ref38] Thomas J. A. Kendall B. J. El-Serag H. B. Thrift A. P. Macdonald G. A. (2024). Hepatocellular and extrahepatic cancer risk in people with non-alcoholic fatty liver disease. Lancet Gastroenterol. Hepatol. 9, 159–169. doi: 10.1016/S2468-1253(23)00275-3, PMID: 38215780

[ref39] Tulsky D. S. Saklofske D. H. Wilkins C. Weiss L. G. (2001). Development of a general ability index for the Wechsler adult intelligence scale--third edition. Psychol. Assess. 13, 566–571. doi: 10.1037//1040-3590.13.4.566 11793899

[ref40] Wang Y. Sun Z. (2009). Current understanding of klotho. Ageing Res. Rev. 8, 43–51. doi: 10.1016/j.arr.2008.10.002, PMID: 19022406 PMC2637560

[ref41] Wang X. Sun Z. (2010). RNAi silencing of brain klotho potentiates cold-induced elevation of blood pressure via the endothelin pathway. Physiol. Genomics 41, 120–126. doi: 10.1152/physiolgenomics.00192.2009, PMID: 20086041 PMC2853900

[ref42] Weinstein G. Davis-Plourde K. Himali J. J. Zelber-Sagi S. Beiser A. S. Seshadri S. (2019). Non-alcoholic fatty liver disease, liver fibrosis score and cognitive function in middle-aged adults: the Framingham study. Liver Int. 39, 1713–1721. doi: 10.1111/liv.14161, PMID: 31155826 PMC6736704

[ref43] Weinstein G. Zelber-Sagi S. Preis S. R. Beiser A. S. DeCarli C. Speliotes E. K.. (2018). Association of Nonalcoholic Fatty Liver Disease with Lower Brain Volume in healthy middle-aged adults in the Framingham study. JAMA Neurol. 75, 97–104. doi: 10.1001/jamaneurol.2017.3229, PMID: 29159396 PMC5833484

[ref44] Xu Y. Sun Z. (2015). Molecular basis of klotho: from gene to function in aging. Endocr. Rev. 36, 174–193. doi: 10.1210/er.2013-1079, PMID: 25695404 PMC4399270

[ref45] Younossi Z. M. Koenig A. B. Abdelatif D. Fazel Y. Henry L. Wymer M. (2016). Global epidemiology of nonalcoholic fatty liver disease—Meta-analytic assessment of prevalence, incidence, and outcomes. Hepatology 64, 73–84. doi: 10.1002/hep.28431, PMID: 26707365

[ref46] Zhang H. Yu L. Yun G. (2023). Reduced serum levels of klotho are associated with mild cognitive impairment in patients with type 2 diabetes mellitus. Diabetes Metab. Syndr. Obes. 16, 129–137. doi: 10.2147/DMSO.S394099, PMID: 36760583 PMC9851628

[ref47] Zhang J. Zhang A. (2023). Relationships between serum klotho concentrations and cognitive performance among older chronic kidney disease patients with albuminuria in NHANES 2011-2014. Front. Endocrinol. 14:977. doi: 10.3389/fendo.2023.1215977, PMID: 37560310 PMC10407554

[ref48] Zhao Y. Zeng C.-Y. Li X.-H. Yang T.-T. Kuang X. Du J.-R. (2020). Klotho overexpression improves amyloid-β clearance and cognition in the APP/PS1 mouse model of Alzheimer’s disease. Aging Cell 19:e13239. doi: 10.1111/acel.13239, PMID: 32964663 PMC7576297

[ref49] Zhou H.-J. Zeng C.-Y. Yang T.-T. Long F.-Y. Kuang X. Du J.-R. (2018). Lentivirus-mediated klotho up-regulation improves aging-related memory deficits and oxidative stress in senescence-accelerated mouse prone-8 mice. Life Sci. 200, 56–62. doi: 10.1016/j.lfs.2018.03.027, PMID: 29544758

