# Supplementary material for: Association of serum klotho with cognitive function among individuals with nonalcoholic fatty liver disease
Source: Front Aging Neurosci. 2024 Nov 5;16:1487182. doi: 10.3389/fnagi.2024.1487182 (PMC11573754; doi:10.3389/fnagi.2024.1487182)
Supplement: Supplementary file 1 [file Table_1.DOCX]

|  | Serum klotho, pg/ mL | | | |  |
| --- | --- | --- | --- | --- | --- |
| Characteristic | Quartile 1  (≤ 656.6) | Quartile 2  (656.7 - 801.3) | Quartile 3  (801.4 - 983.7) | Quartile 4  (≥ 983.8) | *P*_interaction_ |
| Age, years |  |  |  |  |  |
| < 70 | Ref | 0.79 (-1.61 ,3.2) | 0.34 (-2.28 ,2.95) | 0.28 (-2.43 ,2.99) | 0.461 |
| ≥ 70 | Ref | -0.12 (-2.49 ,2.25) | -0.77 (-3.99 ,2.45) | -0.17 (-2.77 ,2.44) |  |
| Gender |  |  |  |  |  |
| Male | Ref | 0.16 (-2.44 ,2.76) | -0.21 (-2.65 ,2.23) | -0.2 (-3.11 ,2.71) | 0.940 |
| Female | Ref | 0.11 (-1.67 ,1.9) | 0.12 (-2.52 ,2.76) | 0.45 (-1.4 ,2.3) |  |
| Education |  |  |  |  |  |
| ≤ High school | Ref | -0.55 (-2.89 ,1.78) | 0.89 (-1.32 ,3.1) | 0.24 (-1.28 ,1.77) | 0.104 |
| Some college or above | Ref | 0.86 (-1.27 ,2.98) | -0.29 (-2.99 ,2.41) | -0.93 (-3.87 ,2.02) |  |
| FMPIR |  |  |  |  |  |
| ≤ 3.0 | Ref | 0.66 (-1.22 ,2.53) | -0.3 (-2.02 ,1.42) | 0.34 (-1.58 ,2.25) | 0.709 |
| > 3.0 | Ref | -0.53 (-4.55 ,3.49) | -0.35 (-4.6 ,3.89) | -0.89 (-5.87 ,4.09) |  |
| Smoking status |  |  |  |  |  |
| Never | Ref | 0.9 (-1.59 ,3.4) | 0.06 (-2.94 ,3.07) | 0.78 (-1.99 ,3.55) | 0.117 |
| Former or current | Ref | -1.43 (-3.5 ,0.64) | -0.9 (-3.33 ,1.54) | -1.34 (-3.87 ,1.2) |  |
| BMI |  |  |  |  |  |
| < 30.0 | Ref | -0.12 (-3.89 ,3.64) | -1.98 (-5.26 ,1.31) | -0.78 (-4.95 ,3.39) | 0.294 |
| ≥ 30.0 | Ref | -0.03 (-2.02 ,1.96) | 0.53 (-2.04 ,3.1) | 0.23 (-2 ,2.46) |  |
| Diabetes |  |  |  |  |  |
| Yes | Ref | 0.42 (-3.83 ,4.68) | 2.85 (-2.41 ,8.12) | 0.24 (-3.91 ,4.4) | 0.143 |
| No | Ref | -0.32 (-2.31 ,1.67) | -1.34 (-3.48 ,0.8) | -0.68 (-3.08 ,1.72) |  |

Supplement table 1. Associations of serum klotho with IRT score in various subgroups.

Abbreviations: NAFLD, Nonalcoholic fatty liver disease; BMI, body mass index; NHANES, National Health and Nutrition Examination Survey.

Data are presented as β (95% CI). Adjust for age, gender, race, education levels, family income–poverty ratio, smoking status, BMI, Stroke, hypertension, antipsychotic, Depression, eGFR, Diabetes, and TG. The strata variable was not included when stratifying by itself.

Bold text indicates a p-value < 0.05, indicating a statistical difference between the two.

|  | Serum klotho, pg/mL | | | |  |
| --- | --- | --- | --- | --- | --- |
| Characteristic | Quartile 1  (≤ 656.6) | Quartile 2  (656.7 - 801.3) | Quartile 3  (801.4 - 983.7) | Quartile 4  (≥ 983.8) | *P*_interaction_ |
| Age, years |  |  |  |  |  |
| < 70 | Ref | 0.23 (-0.83 ,1.29) | 0.07 (-1.12 ,1.27) | 0.35 (-0.96 ,1.66) | 0.257 |
| ≥ 70 | Ref | -0.28 (-1.55 ,0.99) | -0.3 (-2.09 ,1.49) | -0.77 (-2.18 ,0.65) |  |
| Gender |  |  |  |  |  |
| Male | Ref | 0.22 (-0.76 ,1.21) | -0.18 (-1.27 ,0.91) | 0.09 (-1.43 ,1.6) | 0.930 |
| Female | Ref | -0.1 (-1.27 ,1.07) | 0.22 (-1.23 ,1.66) | 0.31 (-0.65 ,1.28) |  |
| Education |  |  |  |  |  |
| ≤ High school | Ref | -0.21 (-1.53 ,1.1) | 0.72 (-0.41 ,1.85) | 0.41 (-0.6 ,1.42) | **0.039** |
| Some college or above | Ref | 0.07 (-0.92 ,1.06) | -0.55 (-1.83 ,0.73) | -0.61 (-1.92 ,0.69) |  |
| FMPIR |  |  |  |  |  |
| ≤ 3.0 | Ref | -0.13 (-1.21 ,0.94) | 0.13 (-0.64 ,0.9) | 0.37 (-0.7 ,1.45) | 0.779 |
| > 3.0 | Ref | -0.51 (-2.38 ,1.37) | -0.69 (-3.04 ,1.65) | -0.68 (-3.19 ,1.84) |  |
| Smoking status |  |  |  |  |  |
| Never | Ref | 0.16 (-1.32 ,1.64) | 0.09 (-1.72 ,1.89) | 0.53 (-0.96 ,2.01) | 0.434 |
| Former or current | Ref | -0.68 (-1.75 ,0.4) | -0.53 (-1.63 ,0.58) | -0.69 (-2.23 ,0.84) |  |
| BMI |  |  |  |  |  |
| < 30.0 | Ref | -0.63 (-2 ,0.74) | -1.12 (-2.66 ,0.42) | -0.26 (-2.37 ,1.85) | 0.157 |
| ≥ 30.0 | Ref | -0.01 (-1.15 ,1.13) | 0.32 (-0.88 ,1.53) | 0.09 (-1.13 ,1.31) |  |
| Diabetes |  |  |  |  |  |
| Yes | Ref | 0.31 (-1.66 ,2.28) | 1.66 (-0.37 ,3.7) | 0.23 (-1.6 ,2.07) | 0.198 |
| No | Ref | -0.55 (-1.74 ,0.64) | -0.8 (-2.07 ,0.47) | -0.31 (-1.89 ,1.27) |  |

Supplement table 2. Associations of serum klotho with DRT score in various subgroups.

Abbreviations: NAFLD, Nonalcoholic fatty liver disease; BMI, body mass index; NHANES, National Health and Nutrition Examination Survey.

Data are presented as β (95% CI). Adjust for age, gender, race, education levels, family income–poverty ratio, smoking status, BMI, Stroke, hypertension, antipsychotic, Depression, eGFR, Diabetes, and TG. The strata variable was not included when stratifying by itself.

Bold text indicates a p-value < 0.05, indicating a statistical difference between the two.

|  | Serum klotho, pg/mL | | | |  |
| --- | --- | --- | --- | --- | --- |
| Characteristic | Quartile 1  (≤ 656.6) | Quartile 2  (656.7 - 801.3) | Quartile 3  (801.4 - 983.7) | Quartile 4  (≥ 983.8) | *P*_interaction_ |
| Age, years |  |  |  |  |  |
| < 70 | Ref | 0.6 (-1.4 ,2.6) | 1.86 (-1.08 ,4.8) | 1.37 (-1.04 ,3.77) | 0.979 |
| ≥ 70 | Ref | 1.24 (-1.85 ,4.32) | 2.3 (-0.83 ,5.42) | 0.26 (-2.09 ,2.62) |  |
| Gender |  |  |  |  |  |
| Male | Ref | 0.35 (-1.82 ,2.52) | 2.01 (-1.1 ,5.13) | 1.12 (-1.51 ,3.74) | 0.914 |
| Female | Ref | 0.76 (-1.7 ,3.21) | 1.43 (-1.37 ,4.22) | 1.75 (-0.83 ,4.33) |  |
| Education |  |  |  |  |  |
| ≤ High school | Ref | 0.62 (-1.67 ,2.91) | 0.57 (-1.51 ,2.65) | 1.36 (-0.1 ,2.82) | **0.038** |
| Some college or above | Ref | 0.31 (-2.11 ,2.73) | **3.29 (0.28 ,6.31)** | 1.46 (-1.14 ,4.06) |  |
| FMPIR |  |  |  |  |  |
| ≤ 3.0 | Ref | **2.52 (0.08 ,4.96)** | **2.74 (0.35 ,5.14)** | 1.53 (-1.21 ,4.27) | 0.258 |
| > 3.0 | Ref | -1.71 (-5.4 ,1.98) | 0.42 (-3.28 ,4.12) | 0.63 (-2.85 ,4.1) |  |
| Smoking status |  |  |  |  |  |
| Never | Ref | 1.03 (-2.38 ,4.43) | 1.31 (-2.65 ,5.26) | 1.45 (-1.21 ,4.12) | 0.817 |
| Former or current | Ref | -0.11 (-1.74 ,1.52) | 1.78 (-0.49 ,4.04) | 1.19 (-0.37 ,2.75) |  |
| BMI |  |  |  |  |  |
| < 30.0 | Ref | 2.42 (-0.98 ,5.81) | 1.79 (-0.63 ,4.22) | 1.42 (-1.6 ,4.45) | 0.771 |
| ≥ 30.0 | Ref | 0.34 (-1.45 ,2.13) | 1.39 (-0.85 ,3.62) | 1.34 (-0.4 ,3.08) |  |
| Diabetes |  |  |  |  |  |
| Yes | Ref | 2.82 (-0.14 ,5.78) | **4.74 (1.12 ,8.35)** | 0.45 (-3.13 ,4.03) | **0.004** |
| No | Ref | -0.62 (-2.3 ,1.05) | 0.25 (-1.81 ,2.3) | 0.67 (-0.96 ,2.3) |  |

Supplement table 3. Associations of serum klotho with AFT score in various subgroups.

Abbreviations: NAFLD, Nonalcoholic fatty liver disease; BMI, body mass index; NHANES, National Health and Nutrition Examination Survey.

Data are presented as β (95% CI). Adjust for age, gender, race, education levels, family income–poverty ratio, smoking status, BMI, Stroke, hypertension, antipsychotic, Depression, eGFR, Diabetes, and TG. The strata variable was not included when stratifying by itself.

Bold text indicates a p-value < 0.05, indicating a statistical difference between the two.
